# Moving time zones in a flash with light therapy during sleep

**DOI:** 10.1038/s41598-023-41742-w

**Published:** 2023-09-02

**Authors:** Renske Lok, Marisol Duran, Jamie M. Zeitzer

**Affiliations:** 1 Department of Psychiatry and Behavioral Sciences, Stanford University, Stanford, CA 94305 USA; 2 Palo Alto Veterans Institute for Research, Palo Alto, CA 94304 USA; 3 Mental Illness Research Education and Clinical Center, VA Palo Alto Health Care System, Palo Alto, CA 94304 USA

**Keywords:** Neuroscience, Neurophysiology

## Abstract

In humans, exposure to continuous light is typically used to change the timing of the circadian clock. This study examines the efficiency of a sequence of light flashes (“flash therapy”) applied during sleep to shift the clock. Healthy participants (n = 10) took part in two 36-h laboratory stays, receiving a placebo (goggles, no light) during one visit and the intervention (goggles, 2-ms flashes broad-spectrum light for 60 min, delivered every 15 s, starting 30 min after habitual sleep onset) during the other. Circadian phase shift was assessed with changes in salivary dim light melatonin onset (DLMO). Sleep, measured with polysomnography, was analyzed to assess changes in sleep architecture and spectral power. After 1 h of flashes, DLMO showed a substantial delay (1.13 ± 1.27 h) compared to placebo (12 ± 20 min). Two individuals exhibited very large shifts of 6.4 and 3.1 h. There were no substantive differences in sleep architecture, but some evidence for greater instability in sleep. 1 h of flash therapy during sleep evokes large changes in circadian timing, up to 6 h, and does so with only minimal, if any, impact on sleep. Flash therapy may offer a practical option to delay the circadian clock in shift workers and jet travelers.

## Introduction

The human circadian pacemaker, located in the suprachiasmatic nucleus (SCN) of the hypothalamus, regulates daily cycles in activity, hormonal levels, and other physiological variables^1,2^. The primary input of the SCN is light, and light information, received by rods, cones, and intrinsically photosensitive (ipRGC) cells in the retina, is sent to the SCN and other hypothalamic regions via the retinohypothalamic tract^3,4^. Through this circuitry, the circadian pacemaker synchronizes to the external natural or artificially imposed light–dark cycle^5^. When traveling across multiple time zones, that synchrony is temporarily lost. This process, called jet lag, can lead to disrupted sleep and wake, increased daytime sleepiness, irritability, and fatigue^6^.

To combat the negative consequences of jet lag, the human circadian system has to re-entrain endogenous physiological and behavioral rhythms to the new light–dark cycle^7^. This can be accomplished by repeated exposure to natural or artificial light at appropriate times of day^8^. The greatest sensitivity of the SCN to light is during the hours of habitual sleep, such that light early in the sleep period induces robust phase delays, and light late in the sleep period induces significant phase advances^9,10^. As such, the optimal time of light exposure for rapid resynchronization to new time zones can require a decision to disrupt standard sleep patterns or to get light at a suboptimal time. Furthermore, the process of re-entrainment using continuous light–dark exposure is relatively slow and can take up to several days^11^.

One potential solution to this problem is the use of a potent light stimulus that can be administered during sleep—flash therapy. Flash therapy involves exposure to a sequence of light flashes rather than continuous light. It has the potential to be at least twice as effective in phase-delaying the circadian system as compared to equiluminant continuous light, a phenomenon observed in multiple animals, including Drosophila^12^, mice^13^, rats^14^, hamsters^15^ and humans^16–18^. The circadian system is sensitive to lower light intensities when receiving flashes^19^, and preliminary evidence indicates it can be administered to individuals while sleeping without interfering with sleep^18,20^. While a single flash does not robustly influence the timing of the circadian clock^21^, a sequence of flashes may work by taking advantage of an unusual aspect of ipRGC physiology. ipRGC neurons continue to be depolarized for several minutes following cessation of stimulation; thus, a flash of light can initiate a depolarization that outlasts the duration of the light itself. The rods or cones that likely underlie the phototransduction of the light flash to an electrochemical signal can resensitize during the darkness between flashes, increasing the photon capture and extrinsic drive on the ipRGC^19,22^. When administering flash therapy during sleep, the flashes must pass through closed eyelids, which reduces the amount of light striking the cornea in a wavelength-dependent manner, with 86% (at 700 nm) to 97% (below 580 nm) attenuation^23,24^.

As the sensitivity of the SCN to light varies over the course of a typical sleep period, so too does the temporal organization of sleep stages, reflective of the intricate interplay between homeostatic sleep pressure and circadian rhythms^25,26^. The progression of sleep throughout the night occurs in a predictable sequence, with non-rapid eye movement sleep, particularly N3, predominantly occurring at the beginning of the night^27^. During this phase, characterized by the prominence of delta waves in the electroencephalograph, the body is less responsive to external stimuli, reducing the likelihood of awakening due to flashes. This time coincides with the time at which the circadian clock is most sensitive to the phase-delaying impacts of light.

This study aims to determine whether we can induce significant phase changes via exposure to flash therapy during sleep and whether such an exposure significantly impacts sleep architecture either during or after the flash therapy. Our primary hypothesis is that flashes at this time of day can significantly delay the circadian clock (H1, with H0 representing the absence of any phase change). Our secondary hypothesis is that these light flashes will modulate sleep (H1, with H0 signifying unchanged sleep patterns due to the flashes).

## Results

### Circadian phase shift (Δϕ)

**Figure 1:**
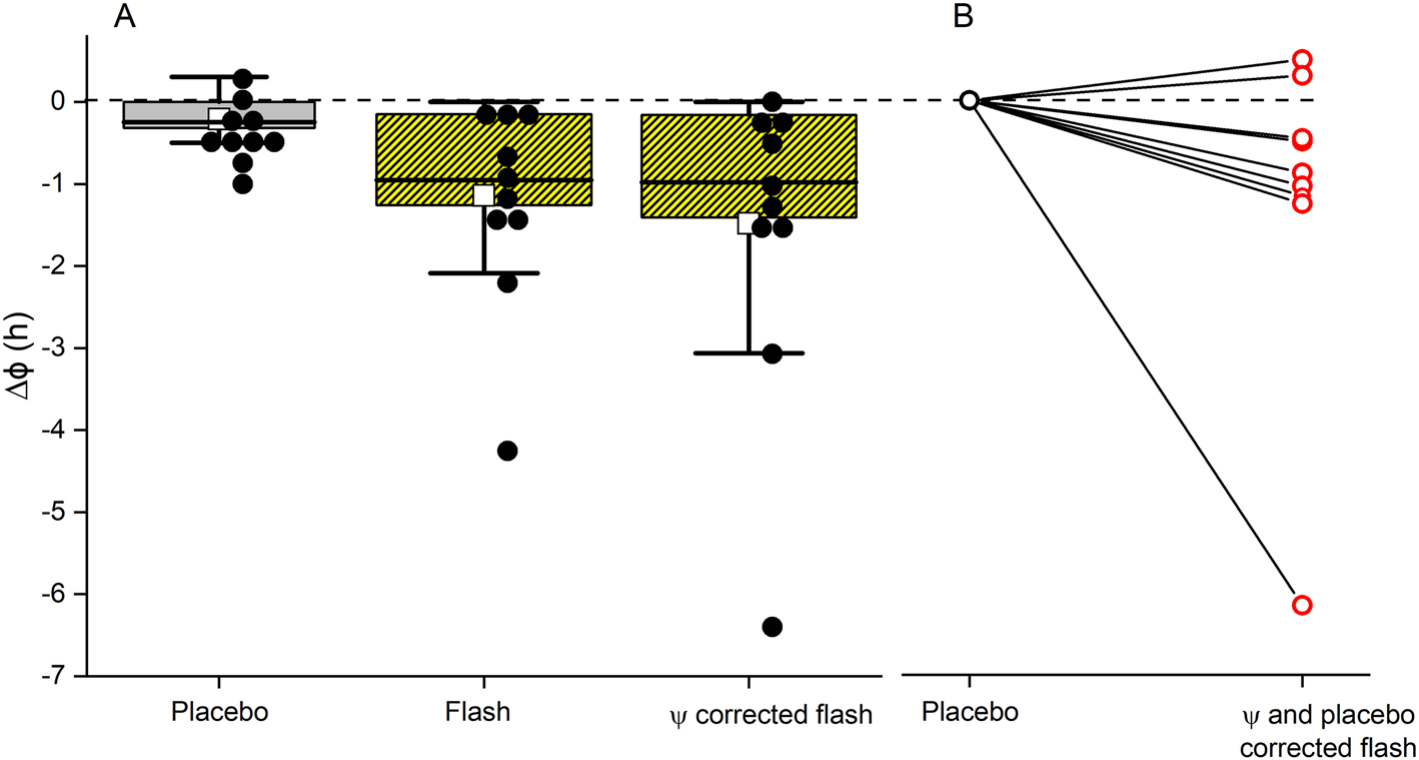
Circadian phase shift (Δϕ). Depicted is the absolute change in melatonin secretion timing (**A**) and the changes relative to baseline melatonin assessment (**B**). A more negative value indicates a greater phase delay. Black dots and red circles indicate individual data points. The phase change presented after placebo, the initially calculated phase change, and the phase change corrected for stimulus timing are shown (**A**).

After exposure to the control condition (no flash therapy but sleeping with goggles), circadian timing showed minimal change (12 ± 20 min). After being exposed to 1 h of flashes, circadian timing showed a substantial change (1.13 ± 1.27 h, Fig. 1). This difference in circadian timing between the conditions was significant (F_(1,20)_ = 6.44, *p* < 0.01) with a large effect size (*d* = 1.02). There was considerable variability in response to the stimulus, with two individuals not displaying any phase changes (0 min), three individuals showing a modest phase change (< 1 h), two individuals displaying a moderate phase change (1–2 h) and two individuals displaying very large phase shifts (> 2 h).

Some of the variability in phase shift may be due to the precise timing of the light vis-à-vis the time of the circadian system (phase angle of stimulation, Ψ). This was calculated post hoc*,* and it was determined that the stimulus onset occurred 2.99 ± 1.38 h after melatonin onset. None of the phase angles of light application were considered outliers (*p* > 0.05, Grubbs test). Using a published phase response curve to 1 h of continuous light stimulation^9^, we corrected for the precise phase of light application. As with the phase-uncorrected data, there was a significant difference in circadian timing after receiving placebo or flashes (F_(1,20)_ = 4.97, *p* < 0.05) with a large effect size (*d* = 0.93) (Fig. 1).

### Sleep architecture: during intervention

**Figure 2:**
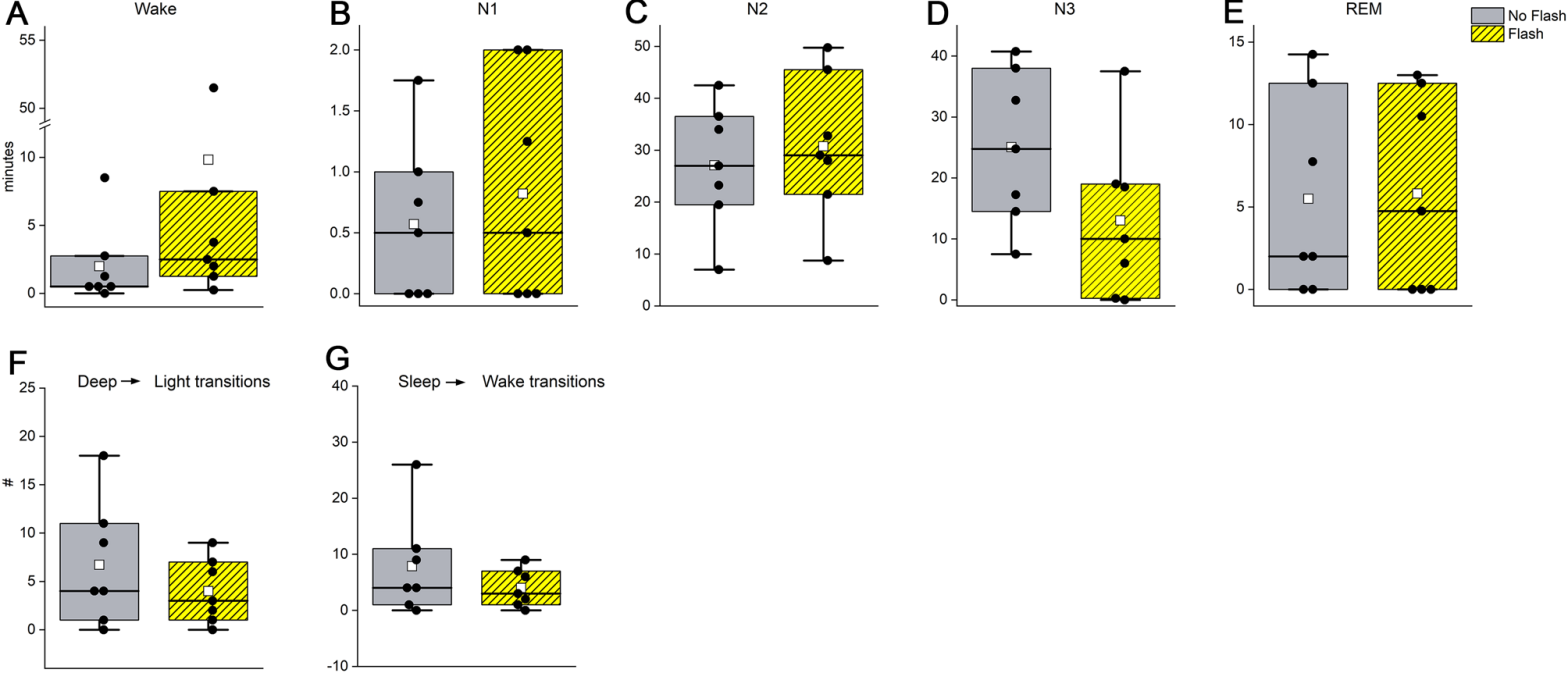
Polysomnography recorded during 1-h of intervention. Presented are the duration spent in each sleep stage during flashes and the same hour during placebo exposure (**A**–**E**), as well as the number of transitions from deep (N3) to light (N2/N1) sleep (**F**) and sleep (REM, N1, N2, N3) to wake transitions (**G**). Placebo (grey) and flash exposure (yellow-black striped) are plotted along with individual data (black dots), the mean (white dots), and the median (black line).

Linear mixed models indicate no significant differences in duration spent in W, N1, N2, N3, or REM sleep during the hour of flash therapy as compared to the same hour during the control condition (F_(4,57)_ = 1.05, *p* = 0.29, Fig. 2A–E). There were also no significant differences in the number of transitions from N2/N3 to N1 sleep (F_(1,13)_ = 1.50, *p* = 0.21, Fig. 2F) or from any stage of sleep (N1, N2, N3, or REM) to wake (F_(1,13)_ = 0.21, *p* = 0.61, Fig. 2G). Inspection of individual hypnograms indicated that one individual had substantially more wake during exposure to the flash sequence (51 min) as compared to the control condition (1 min, Figure S1). This individual, however, was awake *prior* to the onset of the flash sequence. Examination of the other six individual hypnograms did not indicate any substantive W during the flash sequence (Figure S1).

A reverse power analysis, with alpha set to 0.05 and power to 0.80, indicates that given the observed 3.2-min standard deviation in change in total wake duration during the 1-h stimulus (excluding the participant that was already awake at the time of the flashes, Fig. 2), we could have detected a 4.8-min difference in the amount of wake. This suggests that if the flash exposure were to have induced an increase in wake, it would not be greater than 4.8 min.

### Sleep architecture: post-intervention

**Figure 3:**
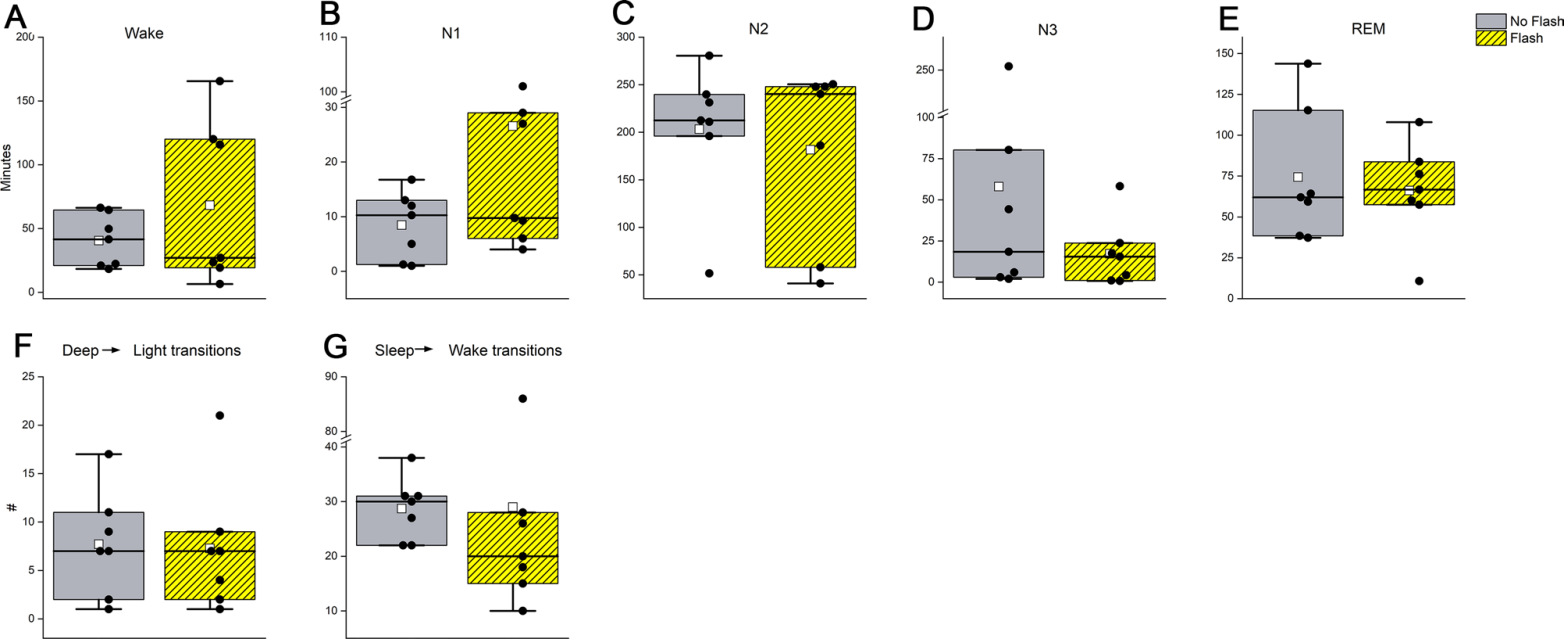
Polysomnographic sleep was recorded during the 6.5 h after the 1-h intervention. Shown is the time spent in each sleep stage following flashes (yellow-black stripe) or placebo (grey) (**A**–**E**), as well as the number of transitions from deep (N3) to light (N2/N1) sleep (**F**) and any stage of sleep (REM, N1, N2, N3) to wake (**G**). Individual data (black dots), mean (white dot), and median (black line) are also presented.

Linear mixed models indicated no significant differences in duration spent in sleep stages during the 6.5 h following the sham or intervention condition (F_(4,57)_ = 1.22, *p* = 0.24, Fig. 3A–E), nor were there differences during this time in the number of transitions from deep to light sleep (F_(1,14)_ = 0.01, *p* = 0.88, Fig. 3F) or sleep to wake (F_(1,14)_ = 0.0009, *p* = 0.97, Fig. 3G; for individual data, see Figure S2).

### Sleep architecture: probability scoring during the intervention

**Figure 4:**
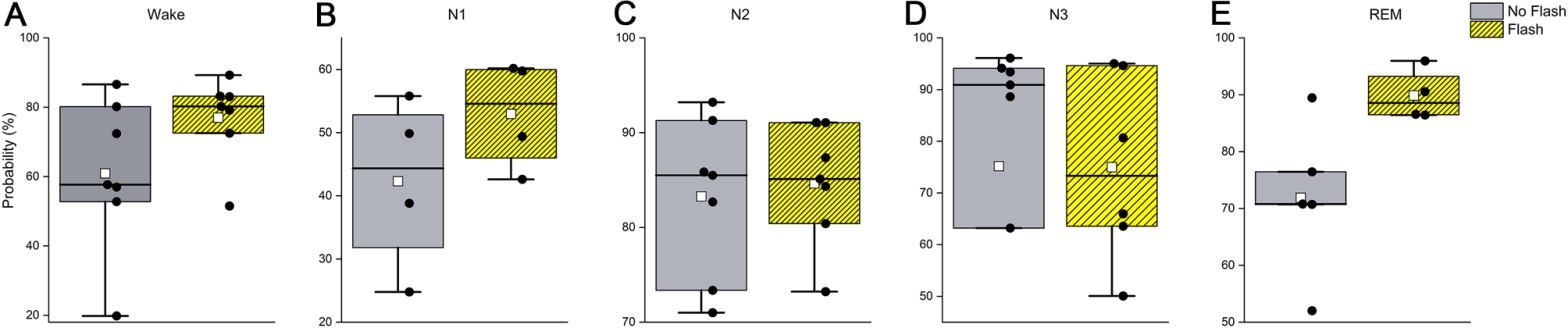
Average probability scoring during flash or placebo therapy. Presented are the average probabilities for wake in 15-s epochs scored as wake (**A**), N1 sleep in 15-s epochs scored as N1 (**B**), N2 sleep in 15-s epochs scored as N2 (**C**), N3 sleep in 15-s epochs scored as N3 (**D**), and rapid eye movement (REM) sleep in 15-s epochs scored as REM (**E**). Placebo (grey) and flash exposure (yellow-black striped) are depicted as individual data (black dots), mean (white dots), and median (black line).

There were no significant differences between flashes and placebo treatment for the probability scoring of wake (F_(1,14)_ = 3.85, *p* = 0.05), N1 (F_(1,8)_ = 1.18, *p* = 0.26), N2 (F_(1,14)_ = 0.23, *p* = 0.60), N3 (F_(1,14)_ = 0.19, *p* = 0.64) or REM sleep (F_(1,9)_ = 1.41, *p* = 0.22) (Fig. 4).

### Sleep architecture: probability scoring post-intervention

**Figure 5:**
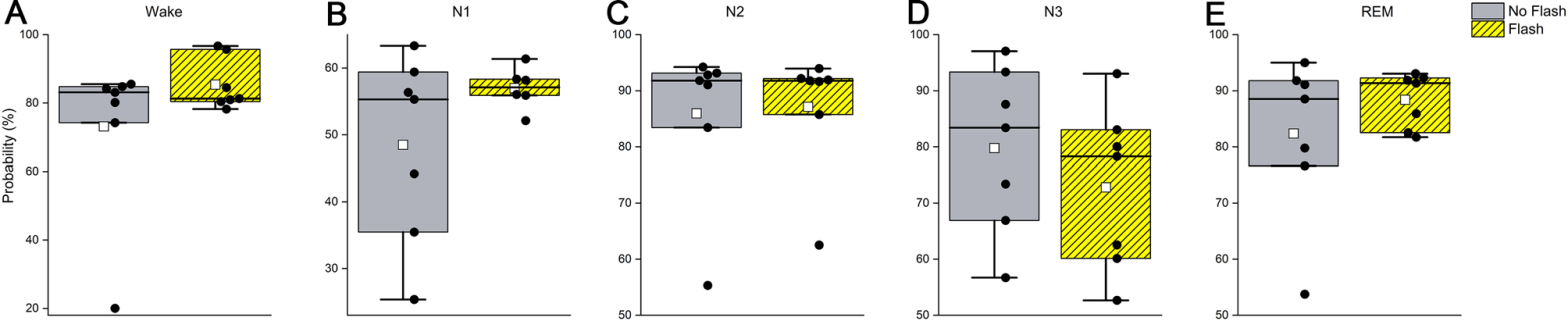
Probability scoring after flash or placebo therapy. Presented are the probabilities for wakefulness (p(W)) (**A**), N1 sleep (p(N1)) (**B**), N2 sleep (p(N2)) (**C**), N3 sleep (p(N3)) (**D**), and rapid eye movement sleep (p(REM)) (**E**) Placebo and flash exposure are depicted in grey and yellow-black striped respectively. Individual data are presented in black, the mean of all data points is indicated in white, and the median is presented as a black line (**B**, **C**).

There were no significant differences between flashes and placebo treatment for the probability scoring of wake (F_(1,14)_ = 1.69, *p* = 0.17), N1 (F_(1,13)_ = 1.69, *p* = 0.17), N2 (F_(1,14)_ = 0.42, *p* = 0.48), N3 (F_(1,14)_ = 0.09, *p* = 0.74), or REM sleep (F_(1,14)_ = 1.42, *p* = 0.22) (Fig. 5).

### EEG spectral power: during intervention

The amount of delta power during flash or placebo exposure significantly differed between conditions (F_(1,60)_ = 8.68, *p* = 0.005), with slightly *more* delta power during flash exposure (*d* = 0.49). The amount of delta power did not significantly differ between conditions depending on sleep stage (wake, N1, N2, N3, REM, F_(4,60)_ = 0.30, *p* = 0.88). There were no significant differences between flashes or placebo treatment for the absolute amount of theta (F_(1,60)_ = 7.62, *p* = 0.02), alpha (F_(1,60)_ = 6.43, *p* = 0.04), sigma (F_(1,60)_ = 1.13, *p* = 0.08), or beta (F_(1,60)_ = 2.14, *p* = 0.07) power. The amount of theta (F_(4,60)_ = 0.34, *p* = 0.88), alpha (F_(4,60)_ = 0.29, *p* = 0.88), sigma (F_(4,60)_ = 0.25, *p* = 0.91), or beta (F_(4,60)_ = 0.34, *p* = 0.85) power did not significantly differ between conditions depending on the sleep stage.

### EEG spectral power: post-intervention

The amount of delta power after flash or placebo exposure did not significantly differ between conditions (F_(1,70)_ = 1.54, *p* = 0.22), nor did the amount of delta power significantly differ between conditions depending on sleep stage (F_(4,70)_ = 0.25, *p* = 0.91). There were no significant differences between flashes and placebo treatment for the amount of theta (F_(1,70)_ = 2.04, *p* = 0.16), alpha (F_(1,70)_ = 2.08, *p* = 0.16), sigma (F_(1,70)_2.10, *p* = 0.15), or beta (F_(1,70)_ = 1.23, *p* = 0.27) power. The amount of theta (F_(4,70)_ = 0.21, *p* = 0.93), alpha (F_(4,70)_ = 0.15, *p* = 0.96), sigma (F_(4,70)_ = 0.12, *p* = 0.98), or beta (F_(4,70)_ = 0.11, *p* = 0.97) or power did not significantly differ between conditions depending on sleep stage.

### The magnitude of phase shift x sleep stage during flash therapy

The interaction between the extent of phase shift relative to the sleep stage during which the flash therapy occurred was non-significant (F_3,23_, = 0.09, *p* = 0.81), suggesting that the magnitude of phase shift did not depend on sleep stage or total sleep duration during light flash exposure. This relationship did not change by removing the participant who was awake for the majority of flash therapy (F_3,21_, = 0.05, *p* = 0.94). Phase shift effect size also did not changed based on the amount of wake during flash therapy (F_1,6_ = 1.31, *p* = 0.32).

### Bayesian analysis of sleep

While frequentist statistics indicated no significant difference in sleep stages or spectral power due to the flash therapy intervention, we also examined these changes using a Bayesian framework. These analyses, for the most part, supported the linear models we employed, with most of the Bayes Factors (*B)* providing little or anecdotal support for H1 (i.e., *B* < 3) or support for H0 (i.e., *B* < 1). During the 6.5 h after the intervention, anecdotal evidence indicated the absence of an effect on delta (B = 0.46), whereas the levels of theta (B = 0.03), alpha (B = 0.02), sigma (B = 0.02), and beta (B = 0.02) power provided moderate evidence for H0.There was moderate evidence for more transitions between sleep and wake both during the flash therapy (*B* = 9.02) and the 6.5 h following it (*B* = 4.11). Similarly, there was moderate evidence for more transitions between N2/N3 and N1 sleep both during the flash therapy (*B* = 4.06) and the 6.5 h following it (*B* = 8.30).

## Discussion

Our study demonstrates that exposure to a sequence of light flashes for 1 h, timed at the beginning of sleep, can phase delay the circadian clock 1.13 ± 1.27 h on average, though ranging from 0 to 6.15 h. Polysomnography records suggested that no substantive differences occurred in sleep micro- or macro-architecture during or following flash therapy. There was, however, evidence of an increase in delta power during the light flashes; this was likely capturing an event-related potential. Bayesian assessment of sleep structure provided some evidence for a difference in the number of shifts from deeper to lighter stages of sleep and from sleep to wake, both during and following flash therapy compared to the placebo. The largest phase shifts reported here are twice as large compared to previously reported phase delays using 1 hour of flashed light^16^ and three times as large as continuous light of up to 10,000 lx^9^.

The potential efficacy of flash therapy in phase delaying the circadian clock has been explored before^16,17,20^. We used results from these previous studies to derive an optimal stimulus regarding timing, flash duration, frequency, and light intensity. Despite the optimization, we still observe large variability in responses. This is unlikely due to the phase of light application as all participants were exposed to light flashes on average 2.86 h after dim light melatonin onset which, according to the human phase response curve (PRC) to 1 h of continuous light^9^, should be the time at which maximal phase delays are observed. Statistically, the dispersion of the phase of light application were similar and when we adjust the magnitude of the phase shift for the PRC, we still observe significant variability in responses. While it is possible that the PRC to flashed light is different from the PRC to continuous light, this is more likely to be in the magnitude rather than the shape of the curve. This type of highly variable response to flashed light stimulus is consistent with that which has been reported in other species, including rodents and Drosophila^12–15^. Given the divergence of the neural circuitry of circadian photoreception in these specifies, especially invertebrates compared to vertebrates, the variability therefore may be intrinsic to the probabilistic nature of photoreception, converting an electromagnetic signal into an electrochemical signal. With the brief duration of exposure to each individual light flash, there may be an insufficient number of retinal (ipRGC) circuits activated for some of the flashes. This could lead to discontinuity in the flash signal (i.e., ipRGC could stop signaling continuously), which reduces the impact of the flashes on the circadian clock^16^.

Exposure to a sequence of light flashes during wake can be an aversive experience. Thus, light flashes have previously been used in field studies of individuals while they are asleep^20^. This is also at the time during which the circadian clock is most sensitive to light^9,28^. To be useful in the field, however, the light flashes must not actively interfere with sleep. In one previous study, we found no evidence for a different sequence of light flashes to interfere with PSG-recorded sleep^18^. In the current study, PSG indicated no substantive differences between sham and flash therapy in sleep quality and architecture. We examined PSG using traditional scoring methods, spectral analysis, and sleep state probability analysis. We were unable to observe significant differences in sleep architecture and probability scoring due to the flashes. Despite our potential lack of statistical power to identify subtle changes in flash-induced sleep architecture or power, we did have the capability to detect gross disturbances in sleep, with an accuracy threshold of 4.8 min of wakefulness or more. However, we did find some evidence for small flash-induced changes in the number of transitions from sleep to wake and deep to light sleep, and an enhancement of delta activity in the light flash condition. This delta enhancement is likely secondary to an evoked response rather than a true induction of delta activity^18^. In one participant, there was much more wake during the 1-h light flash exposure, however, this participant was awake prior to the onset of the light flashes and the presence of the light flashes likely made falling asleep very difficult (they did go to sleep after cessation of the flashes). The light flash sequence was administered at the beginning of the sleep period, so under the highest homeostatic pressure, which may have reduced the likelihood of the flashes causing an acute arousal. In one previous field study in adolescents, light flashes were administered at the end of the sleep period, during which there was greatly reduced homeostatic pressure, and yet no self-reported evidence of sleep disruption when the duration of the light flash exposure was limited to 2 h^20^. The impact of light flashes on sleep architecture and spectral power reported here and in previous studies^18^ appears to be relatively modest; nonetheless, it's important to approach the safety assessment of short (1 h) exposure flash therapy with care. Moderate-intensity continuous light (100 lx) given throughout sleep has been shown to potentially have adverse effects on health^29^, while lower intensity (40 lx) given throughout the night can affect sleep architecture and spectral power^30^.

By placing light flashes near the beginning of sleep, the light flashes were predominantly administrated during N2 and N3 sleep, with very little wake, N1, or REM occurring (Fig. 2). With the limited range of sleep stages, we did not observe any direct interaction between sleep stage and the magnitude of phase shift elicited by the light flashes. Sleep can directly impact the electrophysiologic characteristics of the SCN and, in theory, could change the response of the SCN to the light flash stimuli^31^. Experiments with shorter-duration stimuli that are proactively assigned to be administered during specific stages of sleep would be needed to answer this question definitively.

Limitations of real-world implications arise from certain aspects of our study design. Firstly, participants were subjected to a 2-week circadian stabilization period with regular bed and wake times. The regular light exposure that occurs with this type of sleep–wake schedule maximizes the circadian clock's oscillation amplitude. This deliberate setup effectively targeted the phase delay part of the phase response curve. However, without such circadian phase stabilization, achieving precise targeting of the correct phase becomes challenging, potentially leading to smaller delays or potentially even phase advances. Secondly, participants were confined to our human isolation facility during the in-lab portion of our protocol. The evening preceding and the day following flash exposure, individuals were exposed to lighting of 10 lx. Although this was necessary to obtain accurate measurements of the circadian clock, it also resulted in abnormally low light exposure levels during the daytime. Consequently, the lack of everyday light exposure might have increased the measured effect sizes^32^. These limitations underscore the need for further research incorporating more real-world scenarios to understand the practical implications of our findings better. While our study provides valuable insights into the potential benefits of flash exposure for circadian phase-delaying, future investigations with different settings and population groups will strengthen the generalizability of our conclusions. To further ensure utility of flash exposure, a better understanding of the causes of interindividual variation is also critical.

We show that while 1 h of flash therapy through closed eyelids during sleep can change the circadian clock phase 1.13 ± 1.27 h on average, there is substantial variability, and shifts up to 6 h are observed. While it is impossible to rule out any impact of flash therapy on sleep, we did not observe large disturbances in polysomnographically assessed sleep. Flash therapy could offer an effective option to phase delay the circadian clock in those working rapidly rotating shifts or traveling over multiple time zones, especially when exposure to light should optimally occur during the normal hours of sleep.

### Materials and methods

#### Ethics statement

This study and all related procedures were reviewed and approved by the Stanford University Institutional Review Board and followed the principles expressed in the declaration of Helsinki. Participants provided informed consent prior to any procedures.

### Participants

Ten healthy young participants (females, *n* = 5, aged 25.2 years ± 4.7 years; and males, *n* = 5, aged 24.8 years ± 6.3 years) participated in a randomized, within-subject experimental design consisting of two laboratory stays during one of which they received a placebo and the other of which was an intervention. All participants were non-smokers with normal color vision (Ishihara Color Plate Test^33^). Participants were of intermediate chronotype (reduced Morningness-Eveningness Questionnaire^34^, > 27 or < 11 excluded) and did not have evidence of a sleep disorder (Pittsburgh Sleep Quality Index^35^, > 5 excluded) or depression (Center for Epidemiological Studies Depression Scale^36^, > 27 excluded). Participants were not taking any medications that could impact their sleep (including daily antihistamines or antidepressants), nor was there evidence of alcohol use disorder (Alcohol Use Disorders Identification Test^37^ > 19 excluded). All female participants completed laboratory visits during the menstrual phase of their menstrual cycle or during withdrawal bleeding if they were using oral contraceptives.

### Power analysis

The sample size estimation is based on our main outcome, the phase delay in response to a sequence of millisecond flashes of light. We derived the sample size estimate from a within-subject examination of the impact of flashes during sleep on phase shifts in which we observed an effect size of 2.03^17^. The average phase shift during dark exposure was 3.5 ± 7.3 min, while the shift in response to milliseconds of flashes was 45 ± 13 min. We set alpha level at 0.05 and the desired power at 0.80. This allowed us to determine that a sample size of 4 individuals would be sufficient to detect an effect size of 2.77. However, considering that we stimulate at a different time of day, we adjusted our sample size estimate to include 10 individuals per group. This is in line with other studies conducted in our lab^16–18^.

### Protocol

Before each laboratory stay, participants completed 2 weeks of at-home sleep monitoring. During these 2 weeks, participants self-reported daily wake time, bedtime, and sleep latency using an automated texting service (Twilio integrated into REDCap). Objective sleep timing was monitored with wrist-worn actigraphs (Actiwatch2, Philips-Respironics, Bend OR; or Motionlogger, Ambulatory Monitoring, Ardsley NY; or AX3, Axivity, Newcastle, UK). Participants were instructed to get 7–9 h of sleep per night (fixed within participant) and maintain a regular bedtime and wake schedule (± 30 min of self-selected bed and wake times). The sleep logs and actigraphy confirmed compliance with this schedule upon entry to the laboratory. Participants whose sleep or wake times deviated by more than 30 min of their target time two or more times during the 14-day window were rescheduled. All in-lab procedures were individually timed based on the participants’ midpoint of sleep, defined as the midpoint of the average sleep period during the previous 14 days.

One day before entry to the lab, participants were instructed to refrain from caffeine intake, over-the-counter pain medications, and alcohol. During the 14-day circadian stabilization method, participants were also asked to refrain from legal and illegal drugs (including marijuana). Compliance was verified using urinary (IDTC-II 6 Panel Instant Drug Test Card, CLIAwaived, San Diego, CA USA) toxicology screens.

During the 37-h in-laboratory portion of the study, participants stayed in a time isolation suite at the VA Palo Alto Health Care System. The room had an *en suite* bathroom, and the lighting was controlled from outside the room. Ambient room lighting was < 10 lx in the horizontal angle of gaze during all waking hours and < 0.05 lx during sleep. The suite’s walls were coated with a highly reflective white, titanium dioxide-based paint to minimize variance in illumination intensity. There were no time cues within the suite (clocks, television, radio, etc.).

**Figure 6:**
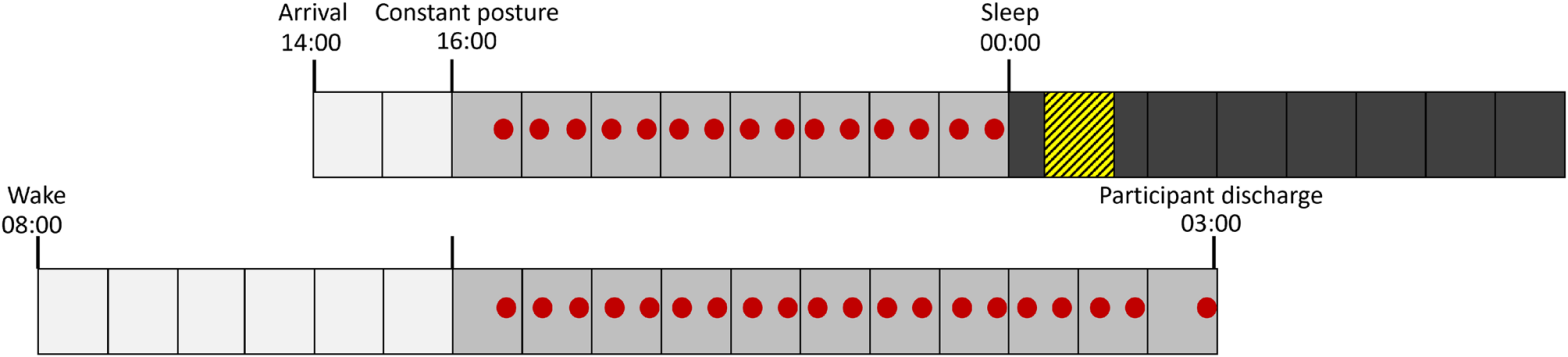
In-laboratory experimental design. Example clock times are given for an individual with HSOn of midnight and HSOff of 8:00; all experimental times were operationalized relative to an individual’s HSOn. An initial and final constant routine (light grey) is used to establish the position of the circadian clock before and after the experimental stimulus (yellow striped) delivered during sleep. Saliva samples (red circles) are collected every half hour.

Participants arrived at the laboratory 7 h after their habitual wake time (HSOff, Fig. 6). From 8 h before habitual sleep onset (HSOn) until HSOn, participants underwent a constant routine^38^ in which they remained in a continuous semi-recumbent position in dim light (< 10 lx in any angle of gaze) and received hourly equicaloric snacks (Ensure original vanilla nutrition shake, Abbott Laboratories, Columbus OH, USA) and equivolumetric water (90 mL) instead of an evening meal, with caloric needs adjusted by individual metabolism and dietary habits^39^. If participants had to urinate or defecate, they remained in bed and were provided with a bedpan or urine bottle. Saliva samples were collected every 30 min by having participants expectorate at least 1 mL of saliva into untreated polypropylene tubes (Fisher Scientific, Pittsburgh, PA), except the last saliva sample, which was collected an hour later due to cognitive testing that took place (described elsewhere). Before sleep, polysomnographic (PSG) electrodes were placed bilaterally over occipital and central locations (O1, O2, C3, C4)^40^, chin (electromyography), and outer canthi (electrooculography), in addition to references (mastoid bone behind the ears) and ground (central forehead) (BWIII EEG Plus, Neurovirtual, Fort Lauderdale FL, USA). Participants were also given custom light-flash emitting goggles^19^ and were asked to keep them on throughout the night. Participants were instructed to keep their eyes closed and try to go back to sleep if they awoke during flash therapy. Room lighting was off during sleep (< 0.05 lx). Thirty minutes after HSOn and lights off, light flashes (specified below) were emitted from the goggles. Technicians observed the participants during light flash administration through an infrared camera system with a hardwired feed outside the room.

Participants were awoken the following day at habitual HSOff. They received a standard hospital breakfast and lunch and were able to move about the time isolation suite. Light in the horizontal angle of gaze was always < 10 lx. Seven hours after HSOff, participants had a second constant posture (as above) that lasted 11 h. After the second constant posture, participants were discharged and provided with a taxi or ride-share service home. After a minimum of 2 weeks with regular sleep (as above), participants returned for a second lab stay under opposite light conditions. The order of light exposure was randomized. The in-lab portion of the protocol took place between January and May, 2022.

### Light exposure

All participants were exposed to an hour-long light stimulus starting 30 min after HSOn. One of the lab visits consisted of wearing custom goggles that were programmed not to operate (protocol control). During the other lab visit, the order of which was randomized, the goggles were programmed to deliver a sequence of light flashes for 1 h. The sequence was composed of 2-ms flashes of broad-spectrum white light delivered every 15 s. Each flash was approximately 1200 photopic lux. The flash parameters’ spectral composition and specificity have been previously reported^19^.

### Data analysis—circadian phase shift

Circadian phase shift was assessed by analyzing the timing of the onset of salivary melatonin on consecutive days in the laboratory, with the experimental stimulus being the only source of light beyond the dim ambient illumination between the two assessments. Salivary melatonin enzyme-linked immunoassay kits (Salimetrics, State College, PA USA; sensitivity = 1.37 pg/mL, range = 0.78–50 pg/mL, intra-assay CV = 5.42%, interassay CV = 8.9%) were used to determine melatonin concentrations per the manufacturer’s instructions. All samples from a single participant were assayed on the same plate.

**Figure 7:**
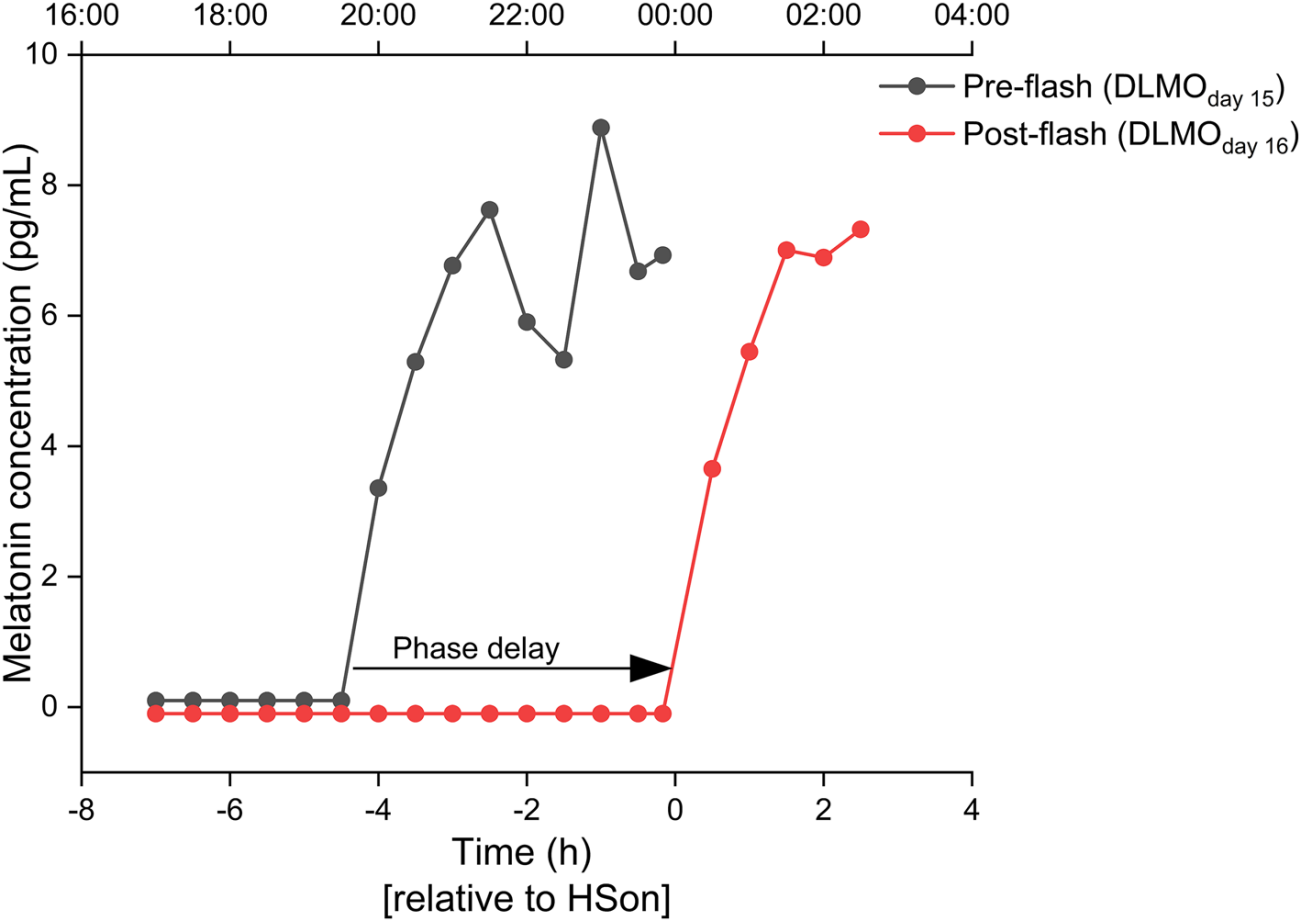
Example of circadian phase delay. Salivary melatonin during baseline (black) and the next day following the stimulus during sleep (red) are indicated.

Melatonin exhibits a near square-wave form, with the onset typically occurring in the hours before habitual bedtime^41^. Melatonin onset was calculated as the time at which salivary melatonin concentrations exceeded a participant-specific threshold, which was calculated as twice the mean of the first three daytime melatonin concentrations (Fig. 7)^42^. Circadian phase shift (Δϕ) was calculated as DLMO _day 15_–DLMO _day 16_ (Fig. 7). The phase angle (θ) between DLMO _day 15_ and the onset of the experimental light exposure was also calculated post hoc to ensure that the participants were exposed to light at a similar circadian phase^9^. Individual deviations from the average phase angle were used to calculate a percentage of change from the average phase shift, which was then applied to each participant’s phase shift to obtain a corrected phase shift, taking into account the differences in circadian timing of the light exposure^16^. The change in phase determined in the control condition (no flashes) was subtracted from phase change after flashes to account for within-individual differences in response to the protocol, notably drift secondary to differences in circadian period length^43–46^.

### Data analysis—sleep polysomnography: sleep architecture

PSG data files were converted to European Data Format and automatically scored using the Stanford-STAGES algorithm (https://github.com/stanford-stages/stanford-stages)^47^, which was developed based on American Academy of Sleep Medicine sleep scoring criteria^48^. In short, the C3, C4, O1, and O2 electroencephalographic channels were extracted from each file as were the bilateral electromyographic chin and electrooculographic channels. Signals were resampled at 100 Hz and filtered between 0.2 and 49 Hz. Each 15-s epoch was scored as either wake (W), N1, N2, N3, or rapid eye movement (REM) sleep. From these data, the duration of each sleep state was calculated, as was the number of deep (stage N3) to light (N2, N1) transitions and sleep (REM, N1, N2, or N3) to wake transitions. As the Stanford-STAGES algorithm is a template matching-based system, the probability of each state was also calculated for each 15-s epoch. For example, a given 15-s epoch could be scored as W, but have a probability distribution of 0.89 W, 0.04 N1, 0.02 N2, 0.00 N3, and 0.05 REM. Another 15-s epoch also scored as W could have a probability distribution of 0.64 W, 0.19 N1, 0.03 N2, 0.00 N3, and 0.14 REM, indicating that the 15-s epoch has a greater mixture of states (i.e., W co-occurring with other states) than the first example. Calculations were performed separately for polysomnography recorded during and after flash therapy. For three individuals, data on one of their visits showed too many artifacts to be analyzed (N = 7 for all polysomnography results).

### Data analysis—sleep polysomnography: spectral power densities

Spectral analysis was performed by applying fast Welch’s Fourier transformation (FFT; hamming, 0% overlapped) on 3-s time windows^49^ (package “rlseep”, version 1.0.6^50^). Artifacts were removed based on sleep stage scoring. Power spectral densities were analyzed for delta (0.5–3.5 Hz), theta (3.5–7.5 Hz), alpha (7.5–13 Hz), sigma (12–14 Hz), and beta (15–30 Hz) power over the central derivations (C3, C4). Power spectral densities were calculated during flash or placebo exposure (1-h interval), post-stimulus (6.5 h), per scored sleep stage (W, N1, N2, N3, or REM).

### Statistics

Linear model fitting was performed in R (R Core Team, version: 4.1.2), using the most recent shell of Rstudio (version: 2021.09) and the “lme4” R-package for mixed-effects modeling. Independent models were constructed to calculate the effects of flash therapy on the circadian clock phase and sleep architecture. Phase change, time spent in each sleep stage, the number of transitions from deep to light and sleep to wake, and the probability scoring of W, N1, N2, N3, or REM were dependent variables. Intervention condition (placebo or flashes) was included as a fixed effect. Participant ID and visit number were included in all models as a random effect. To investigate if the magnitude of phase shift was influenced by sleep stage at the time of flash exposure, a mixed model was created in which phase change was included as the dependent variable, and the interaction between sleep duration and sleep stage was included as a fixed effect. Separate models were created to investigate the effects of flash exposure during sleep (stage N1, N2, N3, or REM) or wake. To investigate differences in power spectral densities, power in the delta, theta, alpha, sigma, and beta bands were added to linear mixed models as dependent variables, with intervention condition (placebo or flashes), and the interaction between intervention condition and sleep stage (as factors) included as a fixed effect. A critical *p*-value of 0.05 was maintained for all statistical circadian phase shift analyses. To correct for multiple testing, Bonferroni correction^51^ was applied for statistical evaluation of flash or placebo exposure on sleep staging (*p* = 0.007) and power spectral density (*p* = 0.01) calculations. Cohen's d was calculated by dividing the mean difference between the flash and control therapy by the standard deviation of that difference. Effect sizes were interpreted as small (d = 0.2), medium (d = 0.5), or large (d = 0.8). To measure the relative evidence for the H1 compared to H0 hypothesis, the Bayes Factor (*B*) was computed (package “BayesFactor”). We employed linear models (“lmBF”), wherein the variation in the dependent factor was assessed under two scenarios: one involving the explanation based on the light condition (full model), and the other involving explanation by random factors (null model). A *B* of 1 indicates the data do not favor either theory more than the other; values between 1 and 4 indicate anecdotal evidence for H1, and *B* between 3 and 10 present moderate evidence for H1^52^. *B* falling within the range of 1/3 to 1 suggest anecdotal evidence, while those within 1/3 to 1/10 suggest moderate evidence, and those within 1/10 to 1/30 suggest strong evidence in favor of H0^53^. All data are presented as mean ± SD.

### Supplementary Information

Supplementary Figures.

## Data Availability

All original data and origin files will be available on the Dryad repository. R-codes are available through the public GitHub repository.
