# Supplementary material for: Moving time zones in a flash with light therapy during sleep
Source: Sci Rep. 2023 Sep 2;13:14458. doi: 10.1038/s41598-023-41742-w (PMC10475014; doi:10.1038/s41598-023-41742-w)
Supplement: Supplementary file 1 — Supplementary Figures. [file 41598_2023_41742_MOESM1_ESM.docx]

**Title:** From Paris to New York in a flash: light therapy during sleep delays the circadian clock up to 6 hours after one night

**Authors:** Renske Lok PhD^1^, Marisol Duran MA ^2^, Jamie M. Zeitzer PhD^1,3^

**Orchid:** Renske Lok: 0000-0003-1684-5625, Jamie Zeitzer: 0000-0001-6174-5282

**Affiliations:**^1^Department of Psychiatry and Behavioral Sciences, Stanford University, Stanford CA 94305, USA
^2^Palo Alto Veterans Institute for Research, Palo Alto, CA 94304, USA
^3^Mental Illness Research Education and Clinical Center, VA Palo Alto Health Care System, Palo Alto CA 94304, USA

**Corresponding author:** Jamie M. Zeitzer, jzeitzer@stanford.edu

Figure S1. Individual sleep architecture data (n=7). Presented are the duration spent in each sleep stage (Wake, N1, N2, N3, REM sleep) during flashes and the same hour during placebo exposure for each of the included individuals (A-G). Placebo (grey) and flash exposure (yellow-black striped) are plotted.

Figure S2. Individual sleep architecture data (n=7). Presented are the duration spent in each sleep stage (Wake, N1, N2, N3, REM sleep) after flashes and the same 6.5 hours during placebo exposure for each of the included individuals (A-G). Placebo (grey) and flash exposure (yellow-black striped) are plotted.
